# Macrophage LRP1 Promotes Diet-Induced Hepatic Inflammation and Metabolic Dysfunction by Modulating Wnt Signaling

**DOI:** 10.1155/2018/7902841

**Published:** 2018-11-04

**Authors:** Dianaly T. Au, Mary Migliorini, Dudley K. Strickland, Selen C. Muratoglu

**Affiliations:** ^1^Center for Vascular and Inflammatory Diseases, University of Maryland School of Medicine, Baltimore, MD 21201, USA; ^2^Department of Surgery, University of Maryland School of Medicine, Baltimore, MD 21201, USA; ^3^Department of Physiology, University of Maryland School of Medicine, Baltimore, MD 21201, USA

## Abstract

Hepatic inflammation is associated with the development of insulin resistance, which can perpetuate the disease state and may increase the risk of metabolic syndrome and diabetes. Despite recent advances, mechanisms linking hepatic inflammation and insulin resistance are still unclear. The low-density lipoprotein receptor-related protein 1 (LRP1) is a large endocytic and signaling receptor that is highly expressed in macrophages, adipocytes, hepatocytes, and vascular smooth muscle cells. To investigate the potential role of macrophage LRP1 in hepatic inflammation and insulin resistance, we conducted experiments using macrophage-specific LRP1-deficient mice (*macLRP1^−/−^*) generated on a low-density lipoprotein receptor knockout (*LDLR^−/−^*) background and fed a Western diet. *LDLR^−/−^; macLRP1^−/−^* mice gained less body weight and had improved glucose tolerance compared to *LDLR^−/−^* mice. Livers from *LDLR^−/−^; macLRP1^−/−^* mice displayed lower levels of gene expression for several inflammatory cytokines, including *Ccl3, Ccl4, Ccl8, Ccr1, Ccr2, Cxcl9,* and *Tnf*, and reduced phosphorylation of GSK3*α* and p38 MAPK proteins. Furthermore, LRP1-deficient peritoneal macrophages displayed altered cholesterol metabolism. Finally, circulating levels of sFRP-5, a potent anti-inflammatory adipokine that functions as a decoy receptor for Wnt5a, were elevated in *LDLR^−/−^; macLRP1^−/−^* mice. Surface plasmon resonance experiments revealed that sFRP-5 is a novel high affinity ligand for LRP1, revealing that LRP1 regulates levels of this inhibitor of Wnt5a-mediated signaling. Collectively, our results suggest that LRP1 expression in macrophages promotes hepatic inflammation and the development of glucose intolerance and insulin resistance by modulating Wnt signaling.

## 1. Introduction

Obesity, dyslipidemia, and insulin resistance are major contributors to the metabolic syndrome (MetS), which can lead to type 2 diabetes (T2D) [1]. It is estimated that 27–29 million people (approximately 9% of the population) in the United States have T2D [2], and consequently, a substantial research effort has been dedicated to elucidating the pathogenesis of diabetes and potential linkages among risk factors. One of the pathways implicated in promoting obesity and inflammation is the Wnt signaling pathway [3–8], which is essential for embryonic development and tissue homeostasis [9]. The large family of secreted Wnt glycoproteins coordinates a multitude of signaling pathways [10].

Canonical Wnt signaling converges on the accumulation and translocation of *β*-catenin into the nucleus. Activation of the pathway occurs when Wnt binds to its receptor frizzled (Fz) and coreceptor low-density lipoprotein receptor-related protein 5/6 (LRP5/6). Seminal work by Ross et al. demonstrated that canonical Wnt signaling regulates adipogenesis and maintains preadipocytes in an undifferentiated state [11]. Proinflammatory cytokines, such as tumor necrosis factor *α* (TNF-*α*) and interleukin 6 (IL-6), produced by adipocytes and/or macrophages, have been shown to promote canonical Wnt signaling and inflammation and inhibit preadipocyte differentiation and lipid accumulation [3, 4]. In contrast to the canonical Wnt signaling pathway, noncanonical Wnt signaling, also known as the *β*-catenin-independent pathway, is less well defined. In this pathway, a noncanonical Wnt ligand, such as Wnt5a, interacts with Fz2 and receptor tyrosine-like orphan receptor 2 (ROR2) to activate Rac1. Wnt5a is expressed in adipocytes and is upregulated in mice on a high fat diet [5] and contributes to obesity-associated inflammation [6–8]. In macrophages, Wnt5a triggers inflammation via activation of c-Jun N-terminal kinase (JNK) signaling pathways [12]. The activity of Wnt5a is regulated by secreted frizzled-related protein 5 (sFRP-5), an extracellular antagonist of Wnt signaling that functions as a decoy receptor by binding Wnt5a and preventing its association with Fz. sFRP-5 is an anti-inflammatory adipokine secreted by adipocytes whose expression is reduced in animal models of metabolic dysfunction [5].

A recent study examining 450 participants in the LIPGENE cohort demonstrated that rs4759277 within the *LRP1* gene is the top single nucleotide polymorphism (SNP) associated with fasting insulin, C-peptide, and insulin resistance [13]. The low-density lipoprotein receptor-related protein 1 (LRP1) is a member of the low-density lipoprotein receptor (LDLR) family and is highly expressed in adipocytes, hepatocytes, macrophages, vascular smooth muscle cells, fibroblasts, and neurons. In addition to its role in the clearance of remnant lipoproteins from the circulation, studies have revealed that LRP1 can bind numerous, unrelated ligands with high affinity and can modulate multiple signaling pathways [14, 15]. We previously reported that macrophage LRP1 contributes to cholesterol accumulation in macrophages, leading to foam cell formation [16]. The current study was undertaken to determine a potential role of macrophage LRP1 in obesity-associated inflammation. Using macrophage-specific LRP1-deficient mice (*macLRP1^−/−^*) generated on an LDLR knockout (*LDLR^−/−^*) background, which were previously used as an insulin resistance model [17], and a high fat, high cholesterol, and high sucrose (Western) diet, we reveal a proinflammatory role of macrophage LRP1 in diet-induced hepatic inflammation through modulation of the Wnt signaling pathway.

## 2. Materials and Methods

### 2.1. Animals

Animal studies were approved by the Institutional Animal Care and Use Committee of University of Maryland School of Medicine. All male mice were weaned at three weeks of age, maintained on a 12-hour light/12-hour dark cycle, fed a standard rodent (chow) diet (Envigo 2018SX) or Western diet (Envigo TD.88137) for the indicated amount of time, and given water ad libitum. Body weights were monitored throughout the experiment and recorded twice per week. Final body weight data are presented as percent body weight gain, calculated as follows:

(1) $$\begin{array}{c} Percent\;Body\;Weight\;Gain=\frac{\left({Weight}_{Final}-{Weight}_{Initial}\right)}{{Weight}_{Initial}}\times 100. \end{array}$$

Embryonic deletion of *lrp1* in macrophages was achieved by crossing *LDLR^+/+^* or *LDLR^−/−^* mice with mice expressing loxP sites flanking the *lrp1* gene (both mice kindly provided by Dr. Joachim Herz at UT Southwestern Medical Center). The resulting offspring, *LDLR^+/+^; lrp1^flox/flox^* and *LDLR^−/−^; lrp1^flox/flox^*, were then crossed with transgenic mice expressing Cre recombinase under the control of a myeloid lineage-specific lysozyme M (LysM) promoter (kindly provided by Dr. Irmgard Förster at the University of Bonn, Germany). Resulting offspring, *lrp1^flox/flox^; LysM-Cre^−/−^* (*LRP1^+/+^*) and *lrp1^flox/flox^; LysM-Cre^+/−^* (*macLRP1^−/−^*) on a *LDLR^+/+^* or *LDLR^−/−^* background, were used in experimental studies with *LRP1^+/+^* littermates, on the appropriate *LDLR* background, serving as controls.

### 2.2. Intraperitoneal Glucose Tolerance Test (IPGTT)

Animals were weighed, transferred to clean cages, and fasted for 4–6 hours. After fasting, baseline (0 minute) blood glucose levels were measured from the tail vein using a Contour USB Blood Glucose Meter (Bayer Healthcare 7393A) and Contour Blood Glucose Test Strips (Bayer Healthcare 7097C). Animals were then administered 1 mg of glucose (100 mg/ml stock glucose concentration; Sigma G-5767) per gram of body weight by intraperitoneal injection, and blood glucose levels were measured at 5, 15, 30, 60, and 120 minutes postinjection. For each animal, blood glucose was plotted against each time point, and the areas under the curve (AUC) and above *y* = 0 were determined using ImageJ (NIH).

### 2.3. Plasma Insulin Determination

Blood was collected from the tail vein during the IPGTT at baseline (0 minute) and 30 and 60 minutes postglucose injection using a heparinized microhematocrit capillary tube (Fisher Scientific 22-362-566). Blood samples were subsequently transferred to a microcentrifuge tube and centrifuged at 1000–2000 ×g for 10 minutes at 4°C, and the supernatant was transferred into a new microcentrifuge tube and stored at −30°C. Plasma insulin levels were quantified using a Rat/Mouse Insulin ELISA Kit (Millipore EZRMI-13K) as per manufacturer's instructions. The plate was washed four times with 1X Wash Buffer (250 *μ*l/well per wash), and the final wash was aspirated. Assay Buffer (10 *μ*l/well) was added to each of the blank and sample wells, and 10 *μ*l of Matrix Solution was added to each of the blank, standard, and control wells. Rat/Mouse Insulin Standards (0.2, 0.5, 1, 2, 5, and 10 ng/ml), Rat/Mouse Insulin Quality Controls 1 and 2, and plasma samples were added to the appropriate wells (10 *μ*l/well) and assayed in duplicate. Detection Antibody (80 *μ*l/well) was added to all wells, and the plate was sealed with a plate sealer and incubated at room temperature for two hours with gentle shaking. After two hours, the plate was washed as described above, and 100 *μ*l of Enzyme Solution was added to each well. The plate was sealed and incubated at room temperature for 30 minutes with gentle shaking. After 30 minutes, the plate was washed six times as described above, and 100 *μ*l of Substrate Solution was added to each well. The plate was incubated at room temperature, protected from light, for 15 minutes with gentle shaking. Stop Solution (100 *μ*l/well) was added, the plate was gently tapped to mix contents, and the plate was read at 410 nm within 5 minutes using a Tecan GENios™ Pro plate reader. Duplicate readings for each blank, standard, and sample were averaged, and the averaged blank optical density (OD) was subtracted. A standard curve was generated by plotting the mean absorbance versus standard concentration for each standard, and a best fit line was determined by linear regression.

### 2.4. Plasma Triglyceride Determination

All blood samples were collected after fasting for 4–6 hours. Blood was collected at 0 weeks (prior to placement on a Western diet) and after 6–7 weeks on a Western diet by tail snip using a heparinized microhematocrit capillary tube and subsequently transferred to a microcentrifuge tube. Blood collection at the experimental endpoint (after 17–18 weeks on a Western diet) was obtained by terminal cardiac puncture using a syringe containing 5 *μ*l of 0.5 M EDTA, pH 8.0 (Invitrogen 15575020), and the blood sample was subsequently transferred to a microcentrifuge tube. Blood collected by tail snip or cardiac puncture was then centrifuged at 1000–2000 ×g for 10 minutes at 4°C. The supernatant was transferred into a new microcentrifuge tube and stored at −30°C. Plasma triglyceride levels were quantified using a Serum Triglyceride Determination Kit (Sigma TR0100). Glycerol standards (0, 3.125, 6.25, 12.5, 25, and 50 *μ*g; Sigma G-7793) and samples were prepared directly in a 96-well microplate, diluted with deionized water, and assayed in duplicate. For all standards and samples, 200 *μ*l of Free Glycerol Reagent was added to each well followed by 50 *μ*l of Triglyceride Reagent. The microplate was incubated at room temperature, protected from light, for 30 minutes, and the absorbance at 550 nm was measured using a Tecan GENios™ Pro plate reader. Duplicate readings for each standard and sample were averaged, and the averaged zero standard OD was subtracted. A standard curve was generated by plotting the mean absorbance versus glycerol amount for each standard, and a best fit line was determined by linear regression.

### 2.5. Quantitative Reverse Transcription Polymerase Chain Reaction (qRT-PCR)

Thioglycollate-elicited peritoneal macrophages or livers were harvested from *LDLR^−/−^* and *LDLR^−/−^; macLRP1^−/−^* mice fed a standard chow or Western diet. Total RNA was isolated using TRIzol™ Reagent (Invitrogen 15596026) as directed by the manufacturer. Total RNA (1 *μ*g) was then used to synthesize cDNA using the RT^2^ First Strand Kit (Qiagen 330401). Real-time PCR was performed on a 7900HT Sequence Detection System (Applied Biosystems) using the RT^2^ SYBR Green ROX qPCR Mastermix (Qiagen 330520) and RT^2^ Profiler™ Mouse Lipoprotein Signaling and Cholesterol Metabolism PCR Array (Qiagen PAMM-080Z) or RT^2^ Profiler™ Mouse Inflammatory Cytokines and Receptors PCR Array (Qiagen PAMM-011Z). Quantitative RT-PCR analyses of *Insr* and *Slc2a2* genes were performed using TaqMan™ Fast Advanced Master Mix (Thermo Fisher Scientific 4444963) and TaqMan® Array Mouse Fatty Liver (Thermo Fisher Scientific 4391524, RAEPRZ3). Relative gene expression data were analyzed using the 2^-ΔΔCt^ method. *Hsp90ab1* was used as a housekeeping gene for data normalization in the Mouse Lipoprotein Signaling and Cholesterol Metabolism PCR Arrays. *Hprt1*, *Hsp90ab1*, and *Gapdh* were used as housekeeping genes for data normalization in the Mouse Inflammatory Cytokines and Receptors PCR Arrays. For the TaqMan® Mouse Fatty Liver Array, *Hprt1*, *Hsp90ab1*, and *Gusb* were used as housekeeping genes for data normalization.

### 2.6. Immunoblotting

Livers were dissected from *LDLR^−/−^* and *LDLR^−/−^; macLRP1^−/−^* mice fed a Western diet for 8 weeks. Each liver was extracted with 150 *μ*l of T-PER™ Tissue Protein Extraction Reagent (Thermo Fisher Scientific) supplemented with cOmplete™, EDTA-free Protease Inhibitor Cocktail (Roche) and Phosphatase Inhibitor Cocktail Set II (EMD Millipore) using a tissue homogenizer (Omni International). Equal amounts of tissue homogenates were separated on a Novex™ 4–12% Tris-Glycine Mini Protein Gel (Invitrogen) and electrophoretically transferred to a nitrocellulose membrane (Thermo Fisher Scientific). The membrane was blocked with 3% Blotting-Grade Blocker (Bio-Rad) and incubated with anti-LRP1 antibody (R2629) [18] at 2.5 *μ*g/ml overnight at 4°C. The membrane was washed three times with 0.05% Tween 20 (Sigma-Aldrich) in tris-buffered saline (TBS-T), and the antibody binding to the membrane was detected with IRDye® 680RD Donkey anti-Rabbit IgG secondary antibody (LI-COR Biosciences) at a concentration of 1 : 5000. The membrane was then washed three times with TBS-T and imaged using a LI-COR Odyssey Infrared Imaging System. Protein levels were quantified by densitometry using ImageJ (NIH) and normalized to Hsp90.

### 2.7. Immunoblot Phosphoprotein Analysis

Immunoblot phosphoprotein analysis was performed by Kinexus Bioinformatics Corporation (Vancouver, British Columbia, Canada) using a Kinetworks™ Phosphosite Screen (Kinexus Bioinformatics Corporation KPSS 1.3 Profiling). Tissues were prepared according to manufacturer's instructions. Briefly, *LDLR^−/−^* and *LDLR^−/−^; macLRP1^−/−^* mice placed on a Western diet for two weeks were euthanized, and the whole animal was perfused with DPBS. The liver was dissected, rinsed in ice-cold DPBS three times, and homogenized in 1 ml of lysis buffer (20 mM MOPS, 2 mM EGTA, 5 mM EDTA, 30 mM NaF, 60 mM *β*-glycerophosphate, 20 mM sodium pyrophosphate, 1 mM Na_3_VO_4_, 1% Nonidet P-40, 1 mM PMSF, 3 mM benzamidine, 5 *μ*M pepstatin A, 10 *μ*M leupeptin, 1 mM DTT, pH 7.2) per 250 mg of liver using a tissue homogenizer (Omni International TH-01). Tissue homogenates were sonicated (Fisher Scientific Sonic Dismembrator Model 100) four times for 10 seconds each time on ice and centrifuged at 90,000 ×g for 30 minutes at 4°C. The supernatant was transferred to a new microcentrifuge tube, and the protein concentration was determined using a Pierce™ BCA Protein Assay Kit. All samples were then prepared in SDS-PAGE sample buffer (31.25 mM Tris-HCl (pH 6.8), 1% (*w*/*v*) SDS, 12.5% (*v*/*v*) glycerol, 0.02% (*w*/*v*) bromophenol blue, and 1.25% (*v*/*v*) *β*-mercaptoethanol) at a final concentration of 1 mg/ml, boiled for four minutes at 100°C, and shipped at room temperature to Kinexus Bioinformatics Corporation. Protein phosphorylation on 38 cell signaling molecules was analyzed using Kinexus KCPS-1.3 phosphoprotein profiling screen software (Kinexus Bioinformatics Corporation). This analysis combines proprietary methodologies with the analytical techniques of gel electrophoresis, immunoblotting, and protein visualization via enhanced chemiluminescence (ECL).

### 2.8. Histological Examination

Livers were dissected from *LDLR^−/−^* and *LDLR^−/−^; macLRP1^−/−^* mice fed a Western diet for 4 weeks. Formalin-fixed and paraffin-embedded livers were analyzed by 5 *μ*m sections. For each liver, sections were subjected to hematoxylin and eosin (H&E) staining to analyze the hepatocyte fat content.

### 2.9. Peritoneal Macrophage Stimulation with Lipopolysaccharide (LPS)

Peritoneal macrophages were harvested from *LDLR^−/−^* and *LDLR^−/−^; macLRP1^−/−^* mice fed a standard chow diet by peritoneal lavage five days after intraperitoneal injection of 1 ml Brewer Modified Thioglycollate broth (3.8% *w*/*v*; BD Biosciences 211716). Macrophages were pooled according to genotype, washed once with Dulbecco's phosphate buffered saline (DPBS; Corning 21-031-CV), and treated with ammonium-chloride-potassium (ACK) lysing buffer for 8 minutes at room temperature. ACK lysing buffer was diluted with Dulbecco's modification of Eagle's medium (DMEM; Corning 10-013-CV) supplemented with 10% fetal bovine serum (FBS; Sigma F-4135), and cells were centrifuged at 1200 rpm for 7 minutes. Macrophages were resuspended in DMEM, 10% FBS, 1X penicillin-streptomycin (P/S; Corning 30-002-CI) (universal culture medium), seeded at 2 × 10^6^ cells per 60 mm tissue culture dish, and maintained at 37°C, 5% CO_2_ in a humidified atmosphere. The following day, nonadherent cells were removed, and adherent cells were washed three times with DPBS and incubated in universal culture medium. After 16–18 hours, cells were washed once with DPBS and serum starved for 16–18 hours. Cells were then washed with DMEM, 1X P/S and treated with or without 50 ng/ml lipopolysaccharide (LPS; Sigma L-2654) in DMEM, 1X P/S for 24 hours. After 24 hours, conditioned media was collected and centrifuged at maximum speed in a microcentrifuge tube for 5 minutes, and the supernatant was transferred to a new microcentrifuge tube and frozen at −30°C. Cells were washed once with ice-cold DPBS and lysed directly on the tissue culture dish with 200 *μ*l/dish of T-PER™ Tissue Protein Extraction Reagent (Thermo Fisher Scientific 78510) supplemented with cOmplete™, EDTA-free Protease Inhibitor Cocktail (Roche 11873580001) and Phosphatase Inhibitor Cocktail Set II (EMD Millipore 524625). Whole cell lysates were transferred to a microcentrifuge tube and centrifuged at maximum speed in a microcentrifuge tube for 5 minutes, and the supernatant was transferred to a new microcentrifuge tube and frozen at −30°C. Lysate protein concentration was determined using a Pierce™ BCA Protein Assay Kit (Thermo Fisher Scientific 23225).

### 2.10. IL-6 Enzyme-Linked Immunosorbent Assay (ELISA)

IL-6 in peritoneal macrophage conditioned media samples was quantified using a LEGEND MAX™ Mouse IL-6 ELISA Kit (BioLegend 431307) as per manufacturer's instructions. The standard was reconstituted as instructed, and diluted standards (0, 3.9, 7.8, 15.6, 31.3, 62.5, 125, and 250 pg/ml) and samples (dilution factor of 1, 50, 100, or 200) were prepared. The plate was washed five times with 1X Wash Buffer (250 *μ*l/well per wash), and the final wash was aspirated. Assay Buffer A (50 *μ*l/well) was added to all wells, and 50 *μ*l of diluted standard or sample was added to each well and assayed in duplicate. The plate was sealed with a plate sealer and incubated at room temperature for two hours with gentle shaking. After two hours, the plate was washed as described above, and 100 *μ*l of Mouse IL-6 Detection Antibody was added to each well. The plate was sealed and incubated at room temperature for one hour with gentle shaking. After one hour, the plate was washed as described above, and 100 *μ*l of Avidin-HRP A solution was added to each well. The plate was sealed and incubated at room temperature for 30 minutes with gentle shaking. After 30 minutes, the plate was washed six times as described above, and 100 *μ*l of Substrate Solution E was added to each well. The plate was incubated at room temperature, protected from light, for 15 minutes. Stop Solution (100 *μ*l/well) was added; the plate was gently tapped to mix contents and was read at 410 nm within 30 minutes using a Tecan GENios™ Pro plate reader. Duplicate readings for each standard and sample were averaged, and the averaged zero standard OD was subtracted. A standard curve was generated by plotting the mean absorbance versus standard concentration for each standard, and a best fit line was determined by linear regression.

### 2.11. sFRP-5 ELISA

Plasma samples were collected as described above, and sFRP-5 in samples were quantified using a Mouse sFRP-5 ELISA Kit (Cloud-Clone Corp. SEC842Mu) as per manufacturer's instructions. The standard was reconstituted as instructed, and diluted standards (0, 0.25, 0.5, 1, 2, 4, 8, and 16 ng/ml) and samples (dilution factor of 10, 20, or 50) were prepared. Standard or sample was added to each well (100 *μ*l/well) and assayed in duplicate, and the plate was sealed with a plate sealer and incubated at 37°C for two hours. After two hours, liquid was aspirated from each well, 100 *μ*l of diluted Detection Reagent A was added to each well, and the plate was sealed and incubated at 37°C for one hour. After one hour, the plate was washed four times with 1X Wash Buffer (250 *μ*l/well per wash). The final wash was aspirated, 100 *μ*l of diluted Detection Reagent B was added to each well, and the plate was sealed and incubated at 37°C for 30 minutes. The plate was washed as described above, the final wash was aspirated, and 90 *μ*l of TMB Substrate Solution was added to each well. The plate was sealed, protected from light, and incubated at 37°C for 15–25 minutes. Stop Solution (50 *μ*l/well) was added, the plate was gently tapped to mix contents, and the plate was read at 410 nm using a Tecan GENios™ Pro plate reader. Duplicate readings for each standard and sample were averaged, and the averaged zero standard OD was subtracted. A standard curve was generated by plotting the mean absorbance versus standard concentration for each standard, and a best fit line was determined by linear regression.

### 2.12. Surface Plasmon Resonance (SPR)

Binding of sFRP-5 (R&D Systems 6266-SF-050) to LRP1 was assessed using a Biacore 3000 optical biosensor system (GE Healthcare Life Sciences). Full-length LRP1, purified from placenta as described [19], was coupled to a Sensor Chip CM5 (GE Healthcare Life Sciences BR-1003-99) using an Amine Coupling Kit (GE Healthcare Life Sciences BR-1000-50). A separate flow cell of the CM5 sensor chip was activated and blocked with 1 M ethanolamine without LRP1 and served as a control surface. Various concentrations of ligand in HBS-P Buffer (0.01 M HEPES, pH 7.4, 0.15 M NaCl, 0.005% (*v*/*v*) Surfactant P20; GE Healthcare Life Sciences BR100368) supplemented with 1 mM CaCl_2_ were flowed over the surface of the LRP1-coupled sensor chip at a rate of 20 *μ*l/min at 25°C. To account for flow cell-dependent refractive index changes, sensorgrams of HBS-P Buffer containing equivalent amounts of buffer in which the protein was prepared were subtracted from the sensorgrams for each of the respective ligand concentrations. Between sample runs, sensor chip surfaces were regenerated with 15 s injections of 100 mM phosphoric acid at a flow rate of 100 *μ*l/min. The data were analyzed by fitting to the observed pseudo-first order rate constant (*k*_obs_) and adding a nonspecific component to determine maximum response units at equilibrium (*R*_eq_). The equilibrium dissociation rate constant (*K*_*D*_) was then determined by plotting *R*_eq_ values versus ligand concentration and fitting the data to a single class of sites using nonlinear regression analysis (GraphPad Prism 7.0 software).

### 2.13. Statistical Analyses

All results are presented as means ± SEM. Data were analyzed for significance using Student's *t*-test or two-way ANOVA followed by Bonferroni posttests. A *p* value < 0.05 was set as the threshold for significance.

## 3. Results

### 3.1. Genetic Deletion of LRP1 in Macrophages Improves Glucose Tolerance and Reduces Body Weight Gain in Western Diet-Fed *LDLR^−/−^* Mice

**Figure 1:**
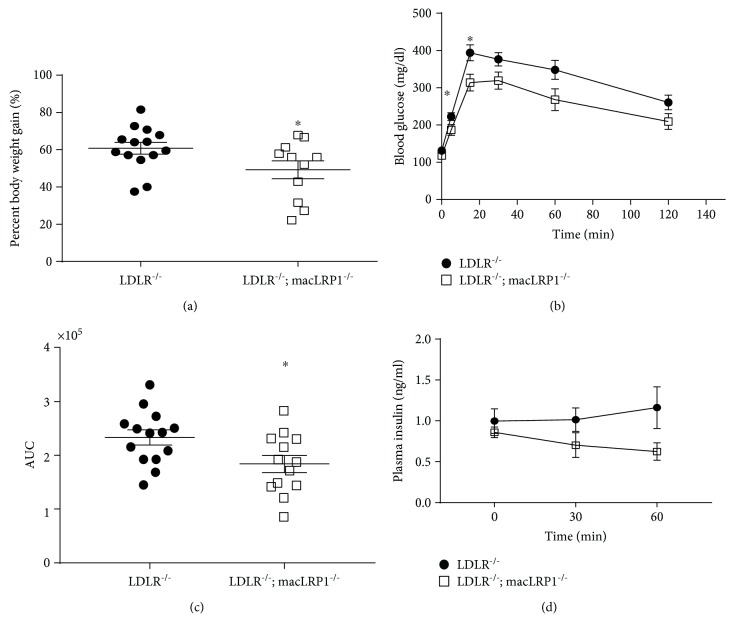
Reduced body weight gain and improved glucose tolerance for *LDLR^−/−^; macLRP1^−/−^* mice. *LDLR^−/−^* and *LDLR^−/−^; macLRP1^−/−^* mice were maintained on a Western diet for 8 weeks prior to conducting the intraperitoneal glucose tolerance test (IPGTT). (a) Percent body weight gain of mice after 8 weeks (*LDLR^−/−^n* = 14, *LDLR^−/−^; macLRP1^−/−^n* = 11). IPGTT (1 mg/g glucose challenge) curve depicting glucose clearance capacity (b) and area under the curve (AUC, c) of *LDLR^−/−^* (*n* = 14) and *LDLR^−/−^; macLRP1^−/−^* (*n* = 13) mice. (d) Plasma insulin levels during the IPGTT (*LDLR^−/−^n* = 8 and *LDLR^−/−^; macLRP1^−/−^n* = 9). The data represent mean ± SEM of results. ^∗^ *p* < 0.05.

To investigate the contribution of macrophage LRP1 to whole body metabolism, *LDLR^−/−^* and *LDLR^−/−^; macLRP1^−/−^* mice were placed on a Western diet for eight weeks and body weights were measured twice per week. *LDLR^−/−^* and *LDLR^−/−^; macLRP1^−/−^* mice demonstrated a similar increasing trend in body weight gain; however, *LDLR^−/−^; macLRP1^−/−^* mice had a significantly smaller gain in percent body weight after eight weeks on a Western diet (Figure 1(a)). We next performed an intraperitoneal glucose tolerance test (IPGTT). Initial fasting blood glucose levels were similar in *LDLR^−/−^* and *LDLR^−/−^; macLRP1^−/−^* mice (Figure 1(b)). However, *LDLR^−/−^; macLRP1^−/−^* mice showed improved glucose tolerance with a lower mean glucose curve (Figure 1(b)) and significantly lower mean area under the curve (AUC) (Figure 1(c)) compared to *LDLR^−/−^* mice. Furthermore, *LDLR^−/−^; macLRP1^−/−^* mice had significantly lower blood glucose levels at 5 minutes and 15 minutes (Figure 1(b)). Plasma insulin levels during the IPGTT were trending lower in *LDLR^−/−^; macLRP1^−/−^* mice; however, differences were not significant (Figure 1(d)).

Interestingly, the impact of LRP1-deficiency in macrophages was only observed in mice on an *LDLR^−/−^* background. When the body weight and glucose metabolism experiments were repeated with *macLRP1^−/−^* mice on an LDLR wild-type (*LDLR^+/+^*) background, no differences were observed between *LDLR^+/+^* and *LDLR^+/+^; macLRP1^−/−^* mice in body weights, fasting glucose levels, glucose tolerance, or plasma triglyceride levels even though the mice were maintained on a Western diet for 17–18 weeks to ensure that any subtle differences attributed to LRP1-deficiency in macrophages would be observed (Figure S1).

### 3.2. Hepatic Inflammation Is Attenuated in *LDLR^−/−^; macLRP1^−/−^* Mice Challenged with a Western Diet

**Figure 2:**
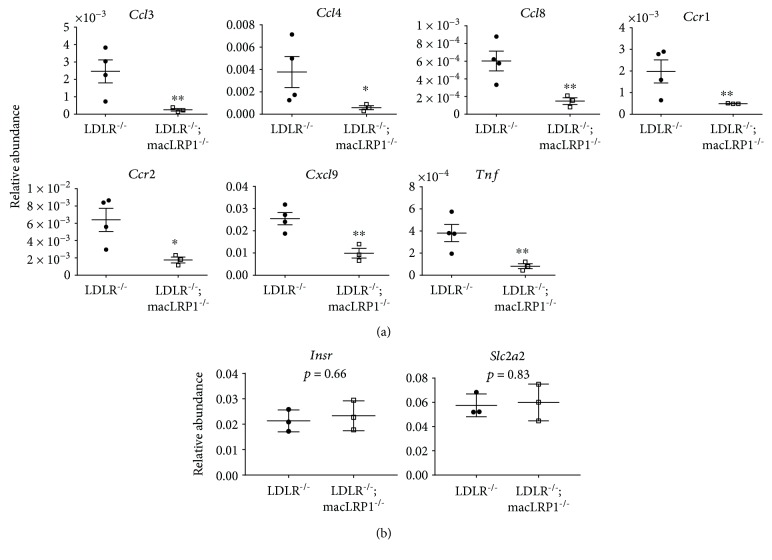
LRP1 expression increased diet-induced liver inflammation in mice. (a) *LDLR^−/−^* and *LDLR^−/−^; macLRP1^−/−^* mice were maintained on a Western diet for 2 weeks. Livers were processed and analyzed for abundance of inflammatory cytokines *Ccl3, Ccl4, Ccl8, Ccr1, Ccr2, Cxcl9,* and *Tnf* by quantitative RT-PCR (*n* = 3). (b) Insulin receptor and Glut2 (*Insr* and *Slc2a2*, respectively) expression levels were analyzed for abundance by quantitative RT-PCR (*n* = 3). The data represent mean ± SEM of results. ^∗^ *p* < 0.05, ^∗∗^ *p* < 0.01.

To further characterize the effects of LRP1 deletion in macrophages, we examined expression levels of several genes involved in mediating the inflammatory response in livers of *LDLR^−/−^* and *LDLR^−/−^; macLRP1^−/−^* mice fed a Western diet for two weeks. Quantitative RT-PCR analyses of livers using a Mouse Inflammatory Cytokines and Receptors PCR Array (Qiagen) revealed seven genes that were significantly downregulated in *LDLR^−/−^; macLRP1^−/−^* mice: *Ccl3*, *Ccl4*, *Ccl8*, *Ccr1*, *Ccr2*, *Cxcl9*, and *Tnf* (Figure 2(a)). We also analyzed expression levels of the insulin receptor (*Insr*) and Glut2 (*Slc2a2*) genes since Ding et al. showed that hepatic LRP1 deletion resulted in marked attenuation of these protein abundances [20]. Quantitative RT-PCR analyses showed no changes in abundances of these genes in livers of *LDLR^−/−^* and *LDLR^−/−^; macLRP1^−/−^* mice fed a Western diet for two weeks (Figure 2(b)).

### 3.3. Regulation of Hepatic Glucose Metabolism Signaling Pathways in *LDLR^−/−^; macLRP1^−/−^* Mice Challenged with a Western Diet

**Figure 3:**
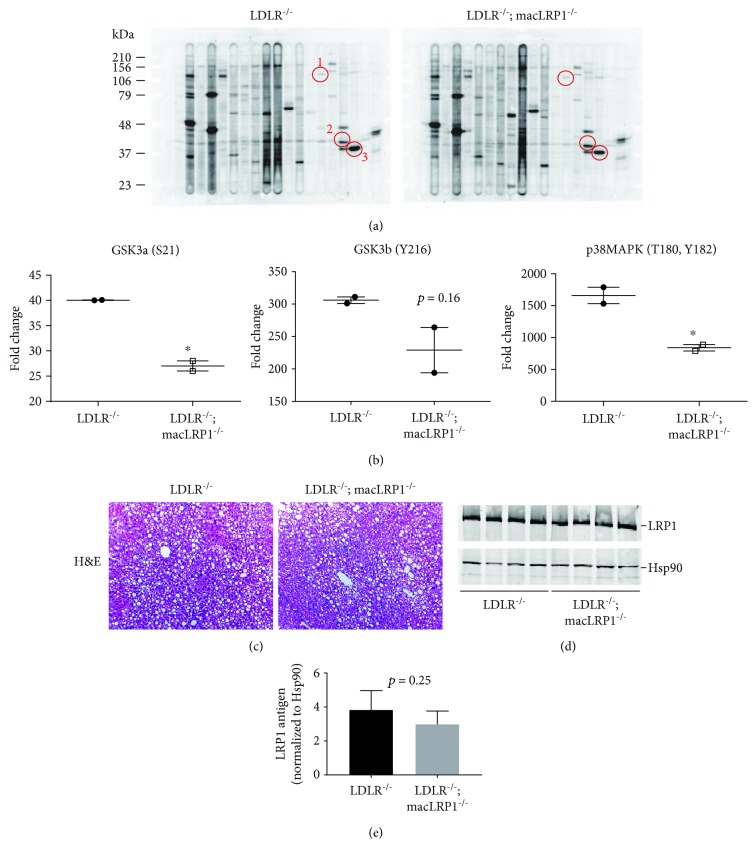
Regulation of hepatic glucose metabolism signaling pathways in *LDLR^−/−^; macLRP1^−/−^* mice challenged with a Western diet. *LDLR^−/−^* and *LDLR^−/−^; macLRP1^−/−^* mice were maintained on a Western diet for 2 weeks. Livers were processed and analyzed for levels of phosphoproteins by Kinexus immunoblot (a, *n* = 2). Molecular weight markers are indicated on the left. Circled bands are specific for GSK3*α* (1), GSK3*β* (2), and p38 MAPK (3). (b) Quantification of immunoblots was performed using Kinexus KCPS-1.3 phosphoprotein profiling screen software. (c) Hepatic fat content was visualized in *LDLR^−/−^* and *LDLR^−/−^; macLRP1^−/−^* mice maintained on a Western diet for 4 weeks by hematoxylin and eosin (H&E) staining. Representative micrographs (20x) of liver sections from *LDLR^−/−^* (*n* = 8) and *LDLR^−/−^; macLRP1^−/−^* (*n* = 9) mice. (d) Hepatic LRP1 expressions in *LDLR^−/−^* and *LDLR^−/−^; macLRP1^−/−^* mice (*n* = 4) that were maintained on a Western diet for 8 weeks. Livers were processed and analyzed for levels of LRP1 by immunoblotting. (e) Immunoblot results in (d) were quantified by densitometry using NIH ImageJ software and normalized to Hsp90 (*p* = 0.25). The data represent mean ± SEM of results. ^∗^ *p* < 0.05.

To complement the inflammation-related gene expression analyses, we also examined activation of several signaling pathways at the protein level in livers of *LDLR^−/−^* and *LDLR^−/−^; macLRP1^−/−^* mice fed a Western diet for two weeks. Mice were placed on a Western diet for two weeks, and livers were processed and analyzed by immunoblot profiling to identify differentially phosphorylated proteins (Figure 3(a), Table S1). Glycogen synthase kinase 3 (GSK3) is a multifunctional kinase that is involved in glucose metabolism and other various physiological pathways. GSK3 has two isoforms (*α* and *β*) that share extensive sequence homology and have similar, but not redundant functions [21]. The activity of this constitutively active serine threonine kinase is regulated by phosphorylation of specific residues. GSK3*α* is inhibited by phosphorylation at serine-21 (S21) by protein kinase B [22], and GSK3*β* is activated by phosphorylation at tyrosine-216 (Y216) [23]. More recently, GSK3*β* activity is reported to be regulated by phosphorylation of the C-terminal residue threonine-390 by p38 MAPK [24]. Phosphoprotein analysis revealed two proteins that were significantly less phosphorylated in livers of *LDLR^−/−^; macLRP1^−/−^* mice, GSK3*α* (S21) and p38 MAPK (T180, Y182), and no change in GSK3*β* (Y216) (Figure 3(b)). This result suggests a unique role for LRP1 in regulating GSK3 function and thereby modulating glucose metabolism. Together, these results suggest that livers from *LDLR^−/−^; macLRP1^−/−^* mice challenged with a Western diet are less proinflammatory and confirm our findings in peritoneal macrophages.

We furthered our investigation on the effects of macrophage LRP1-deficiency on hepatic fat content by analyzing livers of *LDLR^−/−^* and *LDLR^−/−^; macLRP1^−/−^* mice fed a Western diet for four weeks. Hematoxylin and eosin (H&E) staining of liver sections showed no difference in fat accumulation in these livers (Figure 3(c)). Hepatic LRP1 expression was not affected upon macrophage LRP1 deletion as shown by LRP1 immunoblotting of liver extracts from *LDLR^−/−^* and *LDLR^−/−^; macLRP1^−/−^* mice fed a Western diet for eight weeks (Figures 3(d) and 3(e)). This result supports the unaltered hepatic clearance of activated *α*_2_-macroglobulin, a classic LRP1 ligand known to be taken up by hepatocytes [25], by LRP1 in *LDLR^−/−^; macLRP1^−/−^* mice as previously reported [16].

### 3.4. LRP1-Deficient Peritoneal Macrophages Are Less Proinflammatory and Can Modulate Cholesterol Metabolism

**Figure 4:**
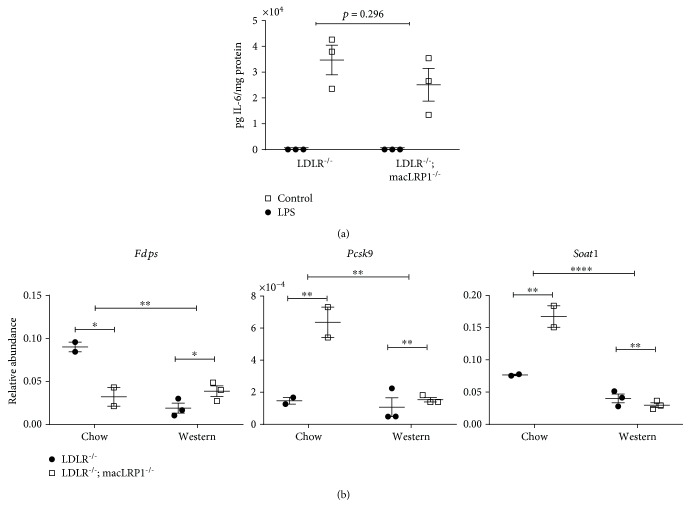
LRP1 modulates the inflammatory response and cholesterol metabolism in macrophages. (a) Thioglycollate-elicited peritoneal macrophages isolated from *LDLR^−/−^* and *LDLR^−/−^; macLRP1^−/−^* mice were challenged with PBS (control) or 50 ng/ml LPS for 24 hours. LPS-induced IL-6 production was measured in the conditioned media (*n* = 3). (b) *LDLR^−/−^* and *LDLR^−/−^; macLRP1^−/−^* mice were maintained on a standard chow or Western diet for 2 weeks, and RNA was isolated from thioglycollate-elicited peritoneal macrophages. *Fdps*, *Pcsk9,* and *Soat1* mRNAs were analyzed by quantitative RT-PCR analyses (chow *n* = 2; Western *n* = 3). The data represent mean ± SEM of results. Two-way ANOVA was used to assess the statistical significance of differences among groups and the effect of a Western diet. ^∗^ *p* < 0.05, ^∗∗^ *p* < 0.01, ^∗∗∗∗^ *p* < 0.0001.

The central roles played by lipoproteins in systemic inflammatory states, such as atherosclerosis, are well established. We decided to further investigate the inflammatory response of macrophages isolated from *LDLR^−/−^* and *LDLR^−/−^*; *macLRP1^−/−^* mice fed a standard chow diet. Thioglycollate-elicited peritoneal macrophages were isolated and treated with PBS (control) or 50 ng/ml LPS for 24 hours, and levels of IL-6 in the conditioned media were measured. LPS-induced IL-6 production in *LDLR^−/−^; macLRP1^−/−^* mice was attenuated compared to *LDLR^−/−^* mice (Figure 4(a)). Although IL-6 levels did not reach significance, this result suggests that LRP1-deficient macrophages may be less proinflammatory when isolated from unchallenged naive mice. To identify a potential role of macrophage LRP1 in lipoprotein metabolism and discern any differences when challenged with a Western diet, we examined expression levels for several genes involved in lipoprotein signaling and cholesterol metabolism. Quantitative RT-PCR analyses of peritoneal macrophages isolated from *LDLR^−/−^* and *LDLR^−/−^; macLRP1^−/−^* mice fed a standard chow or Western diet for two weeks revealed three genes that were differentially regulated: *Fdps*, *Pcsk9*, and *Soat1* (Figure 4(b)). Messenger RNA levels for farnesyl diphosphate synthetase (*Fdps*) (Figure 3(b), left panel), a key enzyme involved in isoprenoid biosynthesis and catalyzes the formation of several metabolic precursors, are significantly downregulated in *LDLR^−/−^; macLRP1^−/−^* peritoneal macrophages isolated from mice fed a chow diet. When placed on a Western diet, however, mRNA levels of *Fdps* significantly dropped in *LDLR^−/−^* peritoneal macrophages compared to those on a chow diet, while levels remained unchanged in *LDLR^−/−^; macLRP1^−/−^* peritoneal macrophages regardless of diet. Messenger RNA levels for proprotein convertase subtilisin/kexin type 9 (*Pcsk9*) (Figure 4(b), center panel), a mediator of plasma cholesterol homeostasis and ligand for LDLR family members, are significantly upregulated in *LDLR^−/−^; macLRP1^−/−^* peritoneal macrophages isolated from mice fed a chow diet. When placed on a Western diet, *Pcsk*9 mRNA levels significantly decreased in *LDLR^−/−^; macLRP1^−/−^* peritoneal macrophages compared to those on a chow diet, while levels remained unchanged in *LDLR^−/−^* peritoneal macrophages regardless of diet. Results similar to *Pcsk9* were also observed for mRNA levels of sterol O-acyltransferase 1 (*Soat1*) (Figure 4(b), right panel), an enzyme localized in the ER and catalyzes the formation of cholesteryl esters. Collectively, these results suggest that circulating LRP1-deficient macrophages on a *LDLR^−/−^* background are less proinflammatory and may influence cholesterol metabolism.

### 3.5. LRP1 Directly Binds to sFRP-5 and May Modulate Wnt Signaling

**Figure 5:**
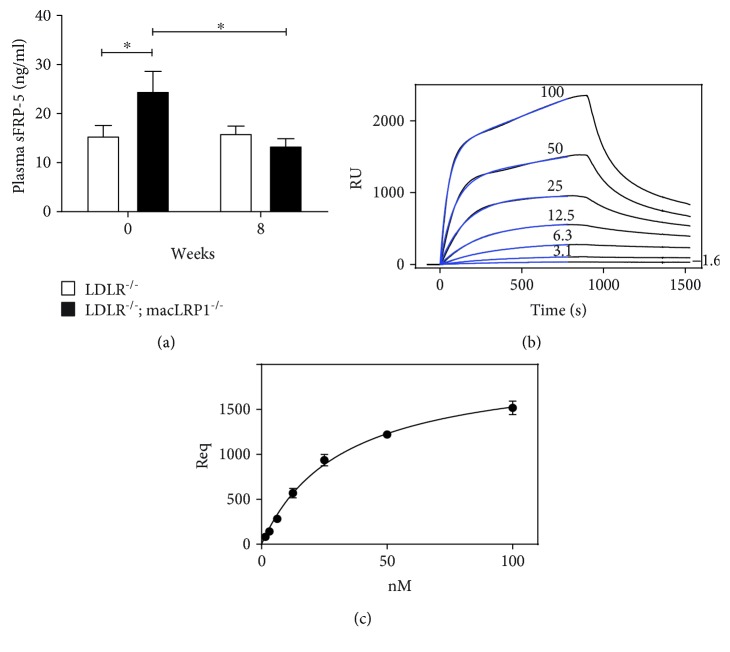
LRP1 directly binds to sFRP-5 and may modulate Wnt signaling. (a) Plasma sFRP-5 levels of *LDLR^−/−^* (*n* = 13) and *LDLR^−/−^; macLRP1^−/−^* (*n* = 11) mice prior to and after being maintained on a Western diet for 8 weeks. (b) Binding of increasing concentrations of sFRP-5 (1.6, 3.1, 6.3, 12.5, 25, 50, and 100 nmol/l) to LRP1 immobilized on the surface of a Biacore sensor chip. (c) *R*_eq_ values, determined from fitting the association data in (b) to a pseudo-first order process with a nonspecific component, were replotted vs. sFRP-5 concentrations, and a *K*_*D*_ value of 32 ± 3 nM was determined by nonlinear regression analysis. The data represent mean ± SEM of three independent experiments. ^∗^ *p* < 0.05.

Noncanonical Wnt signaling has been shown to promote obesity, insulin resistance, and inflammation. Secreted frizzled-related protein (sFRP) family members compete with transmembrane Frizzled receptors for Wnt ligands and can modulate the Wnt signaling pathway. Previous studies conducted by Ouchi et al. showed that secreted frizzled-related protein 5 (sFRP-5) is an anti-inflammatory adipokine that can mediate obesity and metabolic syndrome [5]. To investigate if sFRP-5 is a potential molecular mechanism underlying the improved glucose tolerance, reduced body weight gain, and less proinflammatory phenotype observed in *LDLR^−/−^; macLRP1^−/−^* mice, circulating levels of sFRP-5 were measured in *LDLR^−/−^* and *LDLR^−/−^; macLRP1^−/−^* mice. Prior to placement on a Western diet, *LDLR^−/−^; macLRP1^−/−^* mice had significantly higher levels of baseline plasma sFRP-5 compared to *LDLR^−/−^* mice (Figure 5(a), 0 weeks). After placement on a Western diet for eight weeks, *LDLR^−/−^; macLRP1^−/−^* mice showed a significant reduction in sFRP-5 levels, achieving levels comparable to those measured in *LDLR^−/−^* mice (Figure 5(a), 8 weeks). In contrast, circulating levels of sFRP-5 in *LDLR^−/−^* mice remained unchanged when challenged with a Western diet.

Two members of the LRP family, LRP5 and LRP6, are coreceptors for the Wnt ligand transmembrane receptor Frizzled and can modulate the Wnt/*β*-catenin signaling pathway. Due to the higher circulating levels of sFRP-5 observed in *LDLR^−/−^; macLRP1^−/−^* mice at baseline, binding experiments were performed to test if sFRP-5 directly interacts with LRP1. Surface plasmon resonance (SPR) experiments revealed that sFRP-5 directly binds to purified LRP1 in a concentration-dependent manner (Figure 5(b)). The data were fitted to a pseudo-first order process, adding a nonspecific component to determine maximum response units at equilibrium (*R*_eq_). *R*_eq_ values were then plotted versus concentration to determine a *K*_*D*_ value of 32 ± 3 nM (Figure 5(c)). These results show that LRP1 binds directly to sFRP-5, thereby influencing activation of the noncanonical Wnt signaling pathway.

## 4. Discussion

As a member of the LDL receptor family, a major endocytic function of LRP1 involves clearance of chylomicron remnants from the circulation. Recent studies have revealed an important role for LRP1 in modulating insulin signaling and glucose homeostasis in multiple tissues and suggest a complex interplay with its role in lipoprotein metabolism [26]. In the current study, we used LDLR-deficient mice maintained on a high fat, high cholesterol, and high sucrose (Western) diet, a mouse model that is well suited to investigate liver inflammation in nonalcoholic fatty liver disease [27]. The results of our studies reveal that expression of LRP1 in macrophages promotes hepatic inflammation by a process that appears to involve regulation of the Wnt signaling pathway. First, we demonstrate that *LDLR^−/−^; macLRP1^−/−^* mice have improved glucose tolerance and insulin sensitivity and gained less body weight when fed a Western diet. Second, in conjunction with the improved glucose metabolism, both peritoneal macrophages and livers isolated from *LDLR^−/−^; macLRP1^−/−^* mice produced lower levels of proinflammatory cytokines compared to *LDLR^−/−^* littermates. Third, we discovered that circulating levels of sFRP-5 are elevated in *LDLR^−/−^; macLRP1^−/−^* mice, and fourth, we employed SPR experiments to confirm high affinity binding of sFRP-5 to LRP1, revealing that sFRP-5 is a novel LRP1 ligand. sFRP-5 is a potent anti-inflammatory molecule that modulates metabolic dysfunction in obesity by binding and sequestering Wnt5a preventing its ability to activate the Wnt signaling pathway [11], and regulation of its levels by LRP1 provides a plausible mechanism for the effects we are noting in *LDLR^−/−^; macLRP1^−/−^* mice. Interestingly, an *LDLR^−/−^* background was required to show the effects of *macLRP1^−/−^* on glucose metabolism as our results showed no differences in body weights, fasting glucose levels, glucose tolerance, or plasma triglyceride levels even though the mice were maintained on a Western diet for 17–18 weeks on an *LDLR^+/+^* background. This is likely due to the dramatic increase (>10-fold) in plasma cholesterol and triglyceride levels in mice lacking the LDL receptor when fed a Western diet [28], which is further increased in the *LDLR^−/−^; macLRP1^−/−^* mice [16]. In contrast, the plasma cholesterol and triglyceride levels are only altered about 2-fold in *LDLR^+/+^* mice on a Western diet [28].

**Figure 6:**
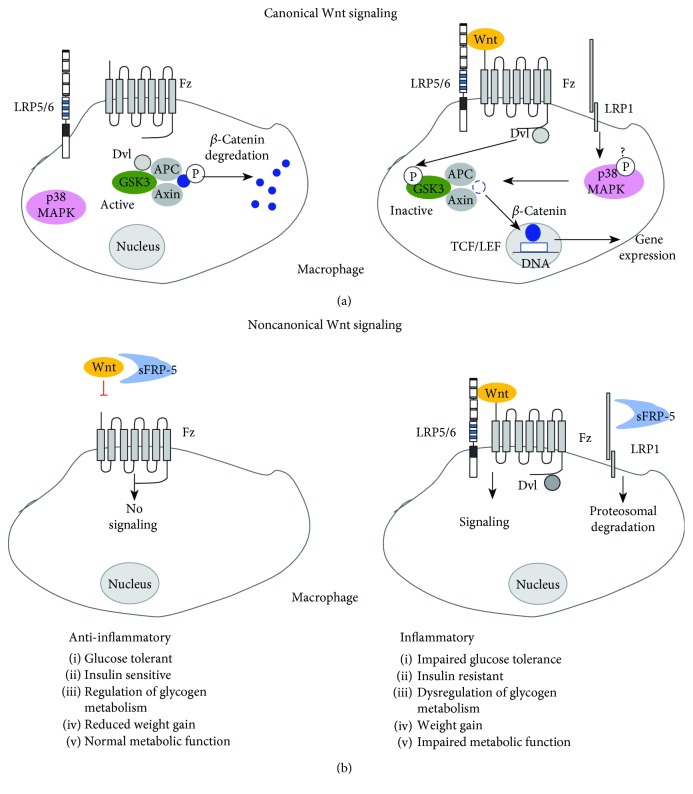
Proposed role of macrophage LRP1 depicted in diet-induced inflammatory conditions. Under proinflammatory conditions, macrophage LRP1 sequesters sFRP-5 to modulate the Wnt signaling pathway and its downstream effectors. LRP1 mediates activation of p38 MAPK which in turn inactivates GSK3. This causes impaired glucose tolerance, dysregulation of glycogen metabolism, and insulin resistance. Altogether, body weight gain with impaired metabolic function is observed in mice. APC: adenomatous polyposis coli; Dvl: dishevelled; Fz: frizzled; sFRP-5: secreted frizzled-related protein 5; GSK3: glycogen synthase kinase 3; LRP1: lipoprotein receptor-related protein 1; LRP5/6: lipoprotein receptor-related protein 5/6; p38 MAPK: p38 mitogen-activated protein kinase; TCF/LEF: T cell factor/lymphoid enhancing factor; Wnt: wingless-related integration site.

Based on our results, we propose a model (Figure 6(b), right panel) where LRP1 modulates the Wnt signaling pathway by directly binding to sFRP-5 and effectively removing this molecule by mediating its cellular uptake and subsequent degradation. As a consequence of removing this inhibitor, Wnt ligands can bind Fz and the coreceptor LRP5/6, resulting in activation of the Wnt signaling pathway. Through unknown mechanisms, our data also revealed that LRP1 expression resulted in increased p38 MAPK activation which would lead to GSK3 inactivation and *β*-catenin accumulation (Figure 6(a), right panel). In contrast, in the absence of LRP1 (Figure 6(b), left panel), sFRP-5 functions as a decoy receptor and sequesters Wnt ligands, and thereby attenuates Wnt signaling resulting in a less proinflammatory state.

Canonical Wnt signaling tightly regulates adipogenesis by maintaining preadipocytes in an undifferentiated state [11]. Independent of its role in adipogenesis, recent advances have demonstrated that Wnt signaling can exert both inflammatory and anti-inflammatory effects in part by modulating the NF-*κ*B pathway [29]. However, the inflammatory roles of Wnt proteins vary widely depending on the tissue- and pathophysiological-specific contexts. Work by Gustafson and Smith showed that both IL-6 and TNF-*α* maintained canonical Wnt signaling and inhibited preadipocyte differentiation and lipid accumulation [3]. Furthermore, IL-6 and TNF-*α* promoted the inflammatory phenotype of adipocytes, providing a link between low-grade inflammation, obesity, insulin resistance, and T2D. Noncanonical Wnt signaling, specifically activation by Wnt5a, has also been shown to promote expression of proinflammatory cytokines in macrophages, which leads to adipose tissue inflammation and impaired glucose homeostasis [7, 8]. Extracellular antagonists of the Wnt signaling pathway prevent ligand-receptor interactions and include Dickkopf (Dkk) family members, Wnt inhibitory factor-1 (WIF-1), Cerberus (CER1), and secreted frizzled-related protein (sFRP) family members [30]. sFRPs bind directly to Wnt, and studies by Ouchi et al. showed that sFRP-5 is an anti-inflammatory adipokine that modulates the adipose tissue macrophage response, glucose tolerance, and hepatic steatosis [5]. Additional studies in human subjects confirmed that lower levels of sFRP-5 are correlated with obesity, impaired glucose tolerance, insulin resistance, and T2D [31, 32]. Collectively, our results suggest that LRP1 may modulate both the canonical and noncanonical Wnt signaling pathways, and further investigation is required to determine the involvement of LRP1 in regulating the levels and function of specific Wnt proteins.

Our results appear to contradict the existing literature demonstrating an atheroprotective role of macrophage LRP1 and its ability to impart an anti-inflammatory effect [33–41]. There are several plausible explanations for the discrepancies observed between the current study and existing literature. First, many of the studies reporting an anti-inflammatory effect of macrophage LRP1 were conducted in mice on a C57BL/6 inbred background. In contrast, our experiments were conducted in mice on a C57BL/6 × 129S6/SvEv mixed background using sibling controls. Multiple studies have reported differences in the inflammatory and metabolic responses to a high fat diet that were dependent on the mouse genetic background [42–45], and it is also likely that a larger variance in the inflammatory and metabolic response is inherent in mice with a mixed background compared to inbred mice. Second, several studies in the existing literature utilize bone marrow transplantation (BMT) to transplant donor *macLRP1^−/−^* bone marrow into irradiated *LDLR^−/−^* recipient mice. While this method is well established for studying hematopoietic cells in the development of atherosclerosis, the known physiological effects of irradiation, such as a lack of body weight gain and gastrointestinal syndrome, render this technique less suitable for studying obesity and T2D [46]. It is also likely that the BMT macrophage response in the atherosclerotic lesion microenvironment will differ from the resident macrophage response in the liver or adipose tissue microenvironment. Finally, modulators of inflammation, such as vascular remodeling induced by carotid artery ligation [47] and the rodent diet composition [48], will affect the macrophage response. Our study utilizes a well-documented genetically engineered mouse strain to study the effects of a representative human Western diet on the macrophage response. This model allows us to examine circulating and tissue-specific resident macrophages in their microenvironment without confounding factors that may alter the immune response.

Another point to consider is the novel dual role of LRP1 in atherosclerosis onset and regression recently reported by Mueller et al. [49]. In this study, the authors demonstrated that LRP1-deficient macrophages directly promote reverse cholesterol transport and transition to an anti-inflammatory phenotype, achieving regression of atherosclerosis caused by a Western diet. This elegant study emphasizes the complex role of LRP1 in both the development and regression of atherosclerosis. In this regard, the altered expressions of *Pcsk9* and *Fdps* in LRP1-deficient peritoneal macrophages in our study are indicative of a more proinflammatory role of LRP1 and support the dual and opposing roles of LRP1 in modulating the inflammatory response. This may explain the attenuated hepatic inflammation in *LDLR^−/−^; macLRP1^−/−^* mice challenged with a Western diet observed in the current study.

Our studies also appear to contradict the protective effect of hepatic LRP1 against hepatic insulin resistance and steatosis [20] and neuronal LRP1 in preventing glucose intolerance in the brain [50]. In the Ding et al. study, inactivation of LRP1 in hepatocytes resulted in obesity, insulin resistance, hyperglycemia, and hepatic steatosis when the mice were placed on a high fat diet. This occurred by suppressing insulin receptor expression and GLUT2 translocation to the plasma membrane. Furthermore, the study found that hepatocyte LRP1 deficiency is associated with enhanced lipogenesis through increased expression of *Scd1* and *Gpd1*. The differences between the Ding et al. study and our current study may be due to excessive lipid uptake in LRP1-expressing macrophages, as we previously demonstrated that LRP1 enhances cholesterol accumulation and foam cell formation in macrophages [16]. Excessive lipid uptake by macrophages is known to trigger a complex sterol-responsive network [51].

Independent of its function in lipoprotein endocytosis, the current study reveals that macrophage LRP1 may also modulate the state of GSK3 activation via multiple pathways. GSK3 is encoded by two different genes which results in two isoforms, *α* and *β*, sharing 97% homology. Unlike other serine/threonine kinases, GSK3 is constitutively active, and phosphorylation of GSK3*α* at Ser21 and GSK3*β* at Ser9 inhibits GSK3 kinase activity [22]. The majority of research on GSK3 function and regulation has been focused primarily on the *β* isoform, and little is known regarding the role of GSK3*α*. Although the isoforms have similar functions, a study by Hoeflich et al. suggested that GSK3*α* is unable to compensate for GSK3*β* gene disruption in murine 129J embryonic stem cells [52]. Studies have also demonstrated that GSK3*α*, but not the *β* isoform, is required for cardiomyocyte survival [53], and GSK3*α* regulates APP processing and the production of A*β* peptides [54] in addition to its role in axon formation [55] and cortical development [56]. Interestingly, Patel et al. found that tissue-specific deletion of GSK3*α* in murine skeletal muscle or liver resulted in no significant differences in glucose or insulin sensitivity compared to wild-type littermates [57]. These results appear to contradict those from Ciaraldi et al. who demonstrated that GSK3*α* knockdown in cultured human skeletal muscle resulted in improved insulin action [58].

In our current study, livers from *LDLR^−/−^; macLRP1^−/−^* mice fed a Western diet for eight weeks had significantly lower levels of phosphorylated GSK3*α* (Ser21) compared to *LDLR^−/−^* littermates, suggesting higher levels of GSK3*α* activity; however, we did not observe any differences in GSK3*β* phosphorylation at Tyr216, which is essential for kinase activity. These results suggest a differential role of GSK3*α* and GSK3*β* in a diet-induced hepatic inflammation model, and GSK3 activity in Kupffer cells, liver resident macrophages, likely contributes to the inflammatory state of the liver. Additional research is needed to discern the overlapping and unique roles of the GSK3 isoforms in different tissue types. Phosphoprotein analysis also revealed significantly lower levels of phosphorylated p38 MAPK (T180, Y182) in *LDLR^−/−^; macLRP1^−/−^* livers. Studies by Thornton et al. demonstrated that p38 MAPK is able to phosphorylate GSK3*β* at the C terminus, resulting in GSK3*β* inactivation and accumulation of *β*-catenin [24]. It is plausible that lower levels of phosphorylated p38 MAPK lead to less GSK3*β* inhibition and increased *β*-catenin degradation, which contribute to the less proinflammatory phenotype observed in *LDLR^−/−^; macLRP1^−/−^* livers. The mechanisms by which macrophage LRP1 modulates p38 MAPK activity are currently unknown, and it is likely that p38 MAPK activity is modulated through the integration of several signaling pathways.

## 5. Conclusions

Taken together, our study suggests that macrophage LRP1 contributes to hepatic inflammation and the development of metabolic dysfunction by modulating activation of GSK3 and levels of sFRP-5 in the Wnt signaling pathways. Our results provide additional insight into the potential mechanisms linking inflammation, metabolic dysfunction, and the development of type 2 diabetes. Further studies are warranted to determine the specific Wnt proteins involved in LRP1-mediated inflammation and if the lipoprotein endocytic function of LRP1 directly affects Wnt signaling. The present study also highlights the complexities of LRP1 in regulating inflammation, stressing that the role of macrophage LRP1 is highly dependent on the tissue microenvironment and in some circumstances may attenuate inflammation, while in others may enhance inflammation.

## Data Availability

All data used to support the findings of this study are included within the article and supplemental materials.

## Supplementary Materials

Supplementary 1 Figure S1: body weight gain and glucose tolerance are similar for *LDLR^+/+^* and *LDLR^+/+^; macLRP1^−/−^* mice. *LDLR^+/+^* and *LDLR^+/+^; macLRP1^−/−^* mice were maintained on a Western diet for 17–18 weeks prior to conducting the intraperitoneal glucose tolerance test (IPGTT). (A) Percent body weight gain of mice after 17–18 weeks (*n* = 7). IPGTT (1 mg/g glucose challenge) curve depicting glucose clearance capacity (B) and area under the curve (AUC, C) of *LDLR^+/+^* (*n* = 7) and *LDLR^+/+^; macLRP1^−/−^* (*n* = 7) mice. (D) Plasma triglyceride levels prior to and after the mice were maintained on a Western diet for 6–7 (*n* = 7) or 17–18 (*n* = 7) weeks. The data represent mean ± SEM of results.

Supplementary 2 Table S1: protein kinases showing an altered phosphorylation state in *LDLR* ^−/−^ and *LDLR* ^−/−^ *; macLRP1* ^−/−^ livers after maintenance on a Western diet for 2 weeks.
