# Supplementary material for: Macrophage LRP1 Promotes Diet-Induced Hepatic Inflammation and Metabolic Dysfunction by Modulating Wnt Signaling
Source: Mediators Inflamm. 2018 Nov 4;2018:7902841. doi: 10.1155/2018/7902841 (PMC6247401; doi:10.1155/2018/7902841)
Supplement: Supplementary 2 — Table S1: protein kinases showing an altered phosphorylation state in LDLR −/− and LDLR −/− ; macLRP1 −/− livers after maintenance on a Western diet for 2 weeks. [file 7902841.f2.docx]

**Table S1.** Protein kinases showing altered phosphorylation state in LDLR^-/-^ and LDLR^-/-^; macLRP1^-/-^ livers after maintenance on a Western diet for 2 weeks.

|  | **Genotype** | | | |  |
| --- | --- | --- | --- | --- | --- |
|  | **Replicate 1** | | **Replicate 2** | |  |
|  | **LDLR^-/-^** | **LDLR^-/-^; macLRP1^-/-^** | **LDLR^-/-^** | **LDLR^-/-^; macLRP1^-/-^** |  |
| **Protein Name** | **Normalized C.P.M.** | | | | **^a^p- value** |
| Adducin alpha (ADD1) [S726] | 313 | 330 | 280 | 306 | 0.207 |
| Adducin gamma (ADD3) [S693] | 223 | 184 | 262 | 198 | 0.548 |
| Src proto-oncogene-encoded protein-tyrosine kinase [Y529] | 1214 | 1368 | 1096 | 1285 | 0.498 |
| Protein-serine kinase C alpha [S657] | 1761 | 1630 | 1305 | 876 | 0.115 |
| Protein-serine kinase C alpha/beta 2 [T638/T641] | 71 | 127 | 99 | 121 | 0.752 |
| MAPK/ERK protein-serine kinase 3/6 (MKK3/6) [S189/S207] | 218 | 171 | 241 | 214 | 0.352 |
| p85 ribosomal protein-serine S6 kinase alpha [T389] | 45 | 42 | 35 | 25 | 0.123 |
| p70 ribosomal protein-serine S6 kinase alpha [T389] | 29 | 30 | 26 | 11 | 0.287 |
| Extracellular regulated protein-serine kinase 1 (p44 MAP kinase) [T202+Y204] | 36 | 36 | 25 | 38 | 0.521 |
| Raf1 proto-oncogene-encoded protein-serine kinase [S259] (63) | 100 | 132 | 124 | 115 | 0.851 |
| Raf1 proto-oncogene-encoded protein-serine kinase [S259] (60) | 454 | 505 | 488 | 545 | 0.439 |
| Protein-serine kinase B alpha [S473] | 38 | 67 | 35 | 31 | 0.316 |
| Glycogen synthase-serine kinase 3 alpha [S21] | 40 | 40 | 26 | 28 | **0.009** |
| Double-stranded RNA-dependent protein-serine kinase [T451] | 52 | 56 | 55 | 64 | 0.328 |
| Glycogen synthase-serine kinase 3 alpha [Y279] | 233 | 240 | 225 | 261 | 0.754 |
| Glycogen synthase-serine kinase 3 beta [Y216] | 301 | 311 | 194 | 264 | 0.165 |
| Retinoblastoma-associated protein 1 [S780] | 46 | 38 | 26 | 49 | 0.720 |
| Mitogen-activated protein-serine kinase p38 alpha [T180+Y182] | 1791 | 1533 | 890 | 790 | **0.027** |
| MAPK/ERK protein-serine kinase 1/2 (MKK1/2) [S218+S222] | 62 | 69 | 54 | 48 | 0.078 |
| cAMP response element binding protein 1 [S133] | 997 | 563 | 728 | 493 | 0.563 |

The letters and numbers in brackets indicate the amino acid residues subject to altered phosphorylation. The numbers in parenthesis indicate observed molecular weight of the protein.

^a^ p-value (LDLR^-/-^ vs. LDLR^-/-^; macLRP1^-/-^ mice, n = 2) calculated by Student’s t-test. p-values < 0.05 are in bold.
